# Supplementary material for: A Deep Learning Neural Network to Classify Obesity Risk in Portuguese Adolescents Based on Physical Fitness Levels and Body Mass Index Percentiles: Insights for National Health Policies
Source: Behav Sci (Basel). 2023 Jun 21;13(7):522. doi: 10.3390/bs13070522 (PMC10376847; doi:10.3390/bs13070522)
Supplement: Supplementary file 1 [file behavsci-13-00522-s001.zip › behavsci-2438492-supplementary materials-Script S1.pdf]

Supplementary material 1

NNET description

# Imported Libraries

```
import keras as k
from keras.models import Sequential
from keras.layers import Dense
import tensorflow
from sklearn.preprocessing import StandardScaler
import pandas as pd
import seaborn as sns
import numpy as np
import seaborn as sns
import matplotlib.pyplot as plt
import sklearn as sk
from sklearn.metrics import confusion_matrix
from sklearn.model_selection import train_test_split
from tensorflow.keras.layers import Dense,Flatten
from sklearn.preprocessing import StandardScaler
from sklearn.metrics import roc_curve
from sklearn.metrics import roc_auc_score
```

# Full script

```
X = new_df.iloc[:,:7].values
y =new_df["Target"].values

X_train,X_test,y_train, y_test = train_test_split(X,y,test_size = 0.3 , random_state = 0)

sc = StandardScaler()
X_train = sc.fit_transform(X_train)
X_test = sc.transform(X_test)

classifier = Sequential()
classifier.add(Dense(activation = "relu", input_dim = 7,
                     units = 4, kernel_initializer = "uniform"))
classifier.add(Dense(activation = "relu", units = 8,
                     kernel_initializer = "uniform"))
classifier.add(Dense(activation = "sigmoid", units = 1,
                     kernel_initializer = "uniform"))
classifier.compile(optimizer = 'adam' , loss = 'binary_crossentropy',
                   metrics = ['accuracy'])

classifier.fit(X_train , y_train , batch_size = 16 ,epochs = 24)

y_pred = classifier.predict(X_test)
y_pred = (y_pred > 0.5)

cm = confusion_matrix(y_test,y_pred)
cm

accuracy = (cm[0][0]+cm[1][1])/(cm[0][1] + cm[1][0] +cm[0][0] +cm[1][1])
print(accuracy*100)

fpr, tpr, thresholds = roc_curve(y_test, y_pred)

roc_auc = roc_auc_score(y_test, y_pred)
roc_auc
```
