# Supplementary material for: A Deep Learning Neural Network to Classify Obesity Risk in Portuguese Adolescents Based on Physical Fitness Levels and Body Mass Index Percentiles: Insights for National Health Policies
Source: Behav Sci (Basel). 2023 Jun 21;13(7):522. doi: 10.3390/bs13070522 (PMC10376847; doi:10.3390/bs13070522)
Supplement: Supplementary file 1 [file behavsci-13-00522-s001.zip › behavsci-2438492-supplementary materials-Script S2.pdf]

Supplementary material 2

K-Fold Cross-Validation description

# Imported Libraries

```
import numpy as np
from sklearn.model_selection import KFold
from sklearn.metrics import accuracy_score, roc_auc_score
from sklearn.svm import SVC
```

# Full script

```
# Assume you have your dataset 'X' and corresponding labels 'y'
# Define your model
model = SVC(probability=True)

# Define the number of folds (K)
k = 10

# Create lists to store the evaluation results
accuracy_scores = []
roc_auc_scores = []

# Create an instance of KFold with the desired number of folds
kf = KFold(n_splits=k, shuffle=True)

# Perform k-fold cross-validation
for train_index, val_index in kf.split(X):
    # Split the data into training and validation sets based on the indices
    X_train, X_val = X[train_index], X[val_index]
    y_train, y_val = y[train_index], y[val_index]

    # Train your model using the training set
    model.fit(X_train, y_train)

    # Make predictions on the validation set
    y_pred = model.predict(X_val)
    y_pred_prob = model.predict_proba(X_val)[:, 1]

    # Calculate the accuracy and ROC AUC score
    accuracy = accuracy_score(y_val, y_pred)
    roc_auc = roc_auc_score(y_val, y_pred_prob)

    # Append the scores to the respective lists
    accuracy_scores.append(accuracy)
    roc_auc_scores.append(roc_auc)

# Compute the average accuracy and ROC AUC score across all folds
avg_accuracy = np.mean(accuracy_scores)
avg_roc_auc = np.mean(roc_auc_scores)
```
